# Magnetic Resonance Angiography Signal Intensity as a Marker of Hemodynamic Impairment in Intracranial Arterial Stenosis

**DOI:** 10.1371/journal.pone.0080124

**Published:** 2013-11-26

**Authors:** Xinyi Leng, Ka Sing Wong, Yannie Soo, Thomas Leung, Xinying Zou, Yongjun Wang, Edward Feldmann, Liping Liu, David S. Liebeskind

**Affiliations:** 1 Department of Medicine and Therapeutics, the Chinese University of Hong Kong, Prince of Wales Hospital, Shatin, Hong Kong Special Administrative Region, China; 2 Department of Neurology, Beijing Tiantan Hospital, Capital Medical University, Beijing, China; 3 Department of Neurology, Tufts Medical Center, Boston, Massachusetts, United States of America; 4 UCLA Stroke Center, University of California Los Angeles, Los Angeles, California, United States of America; Centre Hospitalier Universitaire Vaudois, Switzerland

## Abstract

**Background:**

Intracranial arterial stenosis (ICAS) is the predominant cause of ischemic stroke and transient ischemic attack in Asia. Change of signal intensities (SI) across an ICAS on magnetic resonance angiography (MRA) may reflect its hemodynamic severity.

**Methods:**

In-patients with a symptomatic single ICAS detected on 3D time-of-flight MRA were recruited from 2 hospitals. Baseline and 1-year follow-up data were collected. Signal intensity ratio (SIR) [ =  (mean post-stenotic SI -mean background SI)/(mean pre-stenotic SI - mean background SI)] was evaluated on baseline MRA to represent change of SIs across an ICAS. Acute infarct volume was measured on baseline diffusion-weighted images (DWI). Relationships between SIR and baseline characteristics as well as 1y outcomes were evaluated.

**Results:**

Thirty-six subjects (86.1% males, mean age 55.0) were recruited. Overall, mean SIR was 0.84±0.23. Mean SIRs were not significantly different between the 23 (63.9%) anatomically severe stenoses and the 13 (36.1%) anatomically moderate stenoses (0.80±0.23 versus 0.92±0.21, p = 0.126). SIR was significantly, linearly and negatively correlated to acute infarct volume on DWI (Spearman correlation coefficient −0.471, p = 0.011). Two patients (5.6%) had recurrent ischemic strokes at 1y, not related to SIR values.

**Conclusions:**

Change of signal intensities across an ICAS on MRA may reflect its hemodynamic and functional severity. Future studies are warranted to further verify the relationships between this index and prognosis of patients with symptomatic ICAS.

## Introduction

Intracranial arterial stenosis (ICAS) is the most common cause of ischemic stroke or transient ischemic attack (TIA) in Asian populations, unlike Caucasians [1]. According to previous studies, around 33–50% of ischemic stroke patients and over 50% of TIA patients in China had ICAS [1]–[5]. These patients face a 12–14% risk of recurrent stroke in the subsequent 2 years [6]. In clinical practice, choices concerning treatment for symptomatic patients with ICAS, for instance, medical, interventional or surgical therapy, are usually made according to anatomic severity of ICAS. However, an anatomically significant stenosis might not always possess hemodynamic significance and impaired perfusion. Therefore, evaluating the hemodynamic and functional severity of the stenosis may guide treatment and help identify those at high risk of recurrent stroke.

Time-of-flight (TOF) magnetic resonance angiography (MRA) is a frequently used, noninvasive technique for evaluating intracranial arteries. Based on its contrast mechanism (flow-related enhancement), it also provides information of blood flow features [7]. The degree of enhancement of flowing blood on MRA images, referred to as signal intensity (SI) within the vessel lumen, nonlinearly increases with increasing absolute flow velocity. Turbulence or slow flow would cause signal reduction or even complete signal loss in situ and further downstream in the case of arterial stenosis [8]. Consequently, the change of signal intensity across an arterial stenosis might yield information of its hemodynamic and functional severity.

In a pilot study we developed standard methodology to achieve a feasible and reproducible index termed signal intensity ratio (SIR) to represent the change of SIs across an ICAS on MRA maximum intensity projections (MIP) [9], and we have demonstrated the inter-observer reproducibility of this index in a subsequent study [10]. In this study, we aimed to evaluate relationships between SIR and clinical/imaging factors, and to preliminarily explore relationships between SIR and 1-year outcomes, in stroke patients with symptomatic ICAS.

## Methods

### Ethics Statement

Subjects of the present study were retrospectively collected from the Chinese IntraCranial AtheroSclerosis (CICAS) study. The CICAS study was approved by the ethics committee of the Beijing Tiantan Hospital of Capital Medical University and was conducted according to the principles expressed in the Declaration of Helsinki. All patients or their legal representatives provided written informed consent.

### Subjects

Subjects of this study were retrospectively collected from the CICAS Study, which was a prospective cohort study carried out in 22 hospitals throughout China, enrolling non-cardioembolic ischemic stroke or TIA patients (aged 18–80 years) admitted within 7 days of ictus undergoing brain MRI (including MRA) [11]. TIA in the CICAS study was defined in accordance with the revised definition endorsed by American Heart Association: “a transient episode of neurological dysfunction caused by focal brain, spinal cord, or retinal ischemia, without acute infarction” [12]. Patients recruited to the CICAS study from Beijing Tiantan Hospital (Oct. 2007 to Nov. 2008), Beijing, China and Prince of Wales Hospital (Jun. 2008 to Dec. 2008), Hong Kong SAR, China, were screened. Those with symptomatic single vessel disease (50–99% stenosis, WASID criteria [13]) of anterior circulation [middle cerebral arteries (MCA) or intracranial portion of internal carotid arteries (ICA)] identified by 3D TOF MRA (1.5 or 3.0 Tesla) were enrolled in the present study.

Information about smoking habits, histories of common vascular risk factors and medications, and premorbid modified Rankin Scores (mRS) were collected at admission. Histories of common vascular risk factors were defined as previous diagnosis by the time of admission and/or receiving corresponding medications. Resting blood pressure at admission and results for fasting blood analysis during hospitalization were collected. One-year follow-up was accomplished by phone or by outpatient interview. Primary endpoint referred to recurrent ischemic stroke (fatal or nonfatal) within 1 year, and secondary endpoints included ischemic cardiovascular events (fatal or nonfatal), hemorrhagic stroke (fatal or nonfatal) and death of other causes.

### Brain MRI

Patients were scanned with 1.5 or 3.0 Tesla MR scanners, with sequences as follows: T1- and T2-weighted axial images, fluid attenuated inversion recovery axial images, diffusion-weighted axial images (DWI, slice thickness 5 mm, TR 2500–3500 ms, TE 70–90 ms) and 3D TOF MRA (source images: slice thickness <1.2 mm, TR 20–40 ms, TE 4–7 ms; MIPs: 12 projections; 15° separation). Anatomic severity of stenosis was defined by reviewing radiological reports. Flow void or 70–99% stenosis was regarded anatomically severe, and 50–69% stenosis was regarded anatomically moderate.

**Figure 1:**
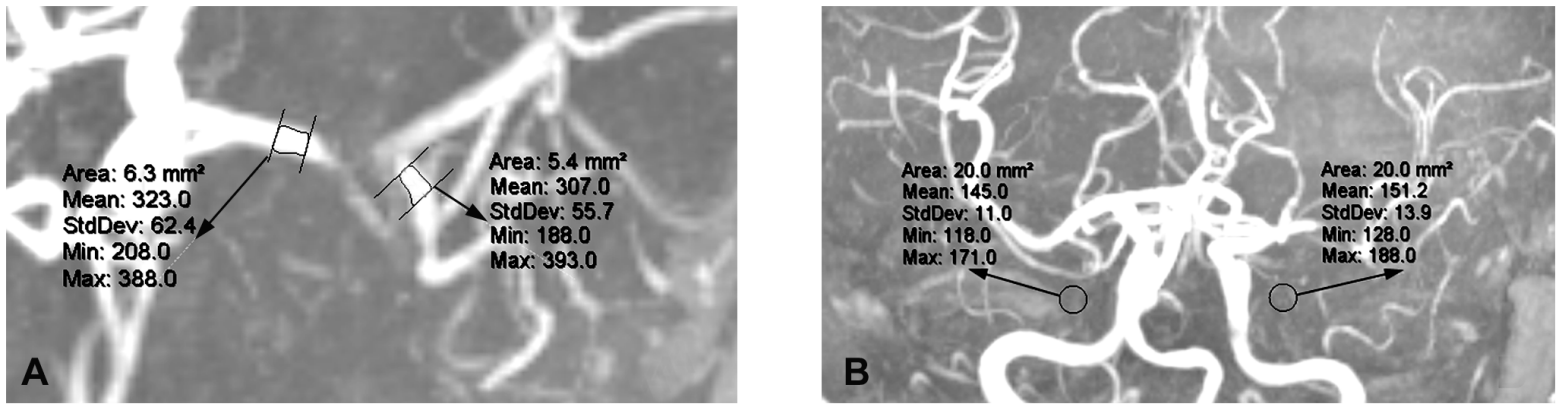
Measurement of signal intensities for a severe stenosis of left middle cerebral artery, using Phillips DICOM viewer R2.6. Mean pre- and post-stenotic signal intensities were 323.0 and 307.0, respectively (A). Mean background signal intensity was the mean of 145.0 and 151.2 (B), being 148.1. Signal intensity ratio was calculated as (307.0–148.1)/(323.0–148.1), which was 0.91.

Evaluation of brain MRI was performed using Phillips DICOM viewer R2.6 (Koninklijke Philips Electronics N.V.). SI measurement on MRA MIPs and SIR calculation were performed by an expert twice, blinded to clinical data. The mean value of repetitive performances was regarded as the SIR value for a single lesion. As developed in our pilot work [9], SIR was calculated as follows, adjusted for background SI: SIR  =  (mean post-stenotic SI - mean background SI)/(mean pre-stenotic SI - mean background SI), where mean pre-stenotic and mean post-stenotic SIs were measured on the MIP showing the greatest degree of stenosis of the target lesion, and mean background SI was the mean value of background SIs within the left and right halves of the anterior-posterior direction MIP. Figure 1 shows the methods for measurement of pre-stenotic, post-stenotic and background SIs.

DWI axial images of diffusion gradient strength b = 1000 were examined by a same reader for acute infarcts at baseline. Volumes of acute infarcts in patients with ischemic stroke were measured twice, 1 month apart from the calculation of SIR and blinded to clinical data. For each case, ischemic lesion(s) on each axial image was outlined, and total infarct volume of this patient (in cm^3^) equaled to the total area of infarcts on all axial images multiplied by slice thickness (5 mm). The mean value of repetitive measurements was regarded as the infarct volume of a single subject.

### Statistical analysis

SIR values were compared between those with anatomically severe or moderate ICAS, and those with or without histories of common vascular risk factors, by using Mann-Whitney test. Spearman correlation coefficient was calculated to evaluate the association between SIR values and acute infarct volumes in patients with ischemic stroke. Also, we performed univariate analyses to evaluate relationships between baseline variables and 1-year outcomes, using Mann-Whitney test for continuous variables and chi-square test for categorical variables. All statistical analyses were performed in PASW Statistics (version 18.0, IBM SPSS Statistics, Chicago, U.S.A.). Two-sided p-values of <0.05 were considered statistically significant.

## Results

### Patient characteristics

Thirty-six subjects (86.1% males, mean age 55.0 years) with symptomatic single vessel disease within the anterior circulation were recruited, among whom 6 and 30 patients underwent 1.5 and 3.0 T MR examinations, respectively. Premorbid mRS scores were all 0–2. Baseline characteristics are shown in Table 1. Numbers of patients with histories of dyslipidemia, hypertension, diabetes mellitus, ischemic heart disease and stroke/TIA were 7 (19.4%), 20 (55.6%), 10 (27.8%), 5 (13.9%) and 7 (19.4%), respectively. None of the subjects had a history of atrial fibrillation. The median interval between symptom onset and admission was 2 days (IQR 1 to 4). The median NIHSS at admission was 3 (IQR 0.25 to 6.75).

**Table 1:** Patient characteristics.

Characteristics a	N = 36
Baseline characteristics	
Age, y	55 (13.7)
Male	31 (86.1)
Current smoker	19 (52.8)
History of dyslipidemia	7 (19.4)
History of hypertension	20 (55.6)
History of diabetes mellitus	10 (27.8)
History of ischemic heart disease	5 (13.9)
History of stroke/transient ischemic attack	7 (19.4)
History of atrial fibrillation	0
Interval from onset to admission, days	2 (1–4)
NIHSS at admission	3 (0.25–6.75)
Systolic blood pressure at admission, mmHg	150.8 (24.1)
Diastolic blood pressure at admission, mmHg	86.9 (10.5)
Fasting blood glucose, mmol/L	5.95 (2.36)
Total cholesterol, mmol/L	4.36 (0.93)
Triglycerides, mmol/L	1.88 (1.22)
High-density lipoprotein, mmol/L	1.06 (0.21)
Low-density lipoprotein, mmol/L	2.67 (0.82)
Brain MRI	
Interval from onset to MRI, days	6 (4–8)
TOF MRA	
Anatomically severe stenoses	23 (63.9)
Anatomically moderate stenoses	13 (36.1)
Signal intensity ratio	0.84 (0.23)
DWI	
Acute infarct detected on DWI	28 (77.8)
Infarct volume, cm^3^	2.40 (0.80–5.78)^b^
1-year follow-up	
Primary endpoint	2 (5.6)
Secondary endpoint	0

NIHSS, National Institutes of Health Stroke Scale; MRI, magnetic resonance imaging; TOF MRA, time-of-flight magnetic resonance angiography; DWI, diffusion-weighted imaging.

a Values are means (SD), medians (IQR) or numbers (%).

b n = 28.

The median interval between symptom onset and MRI examination was 6 days (IQR 4 to 8). Twenty-eight (77.8%) subjects were diagnosed as ischemic stroke, with acute infarct(s) detected on DWI, median infarct volume being 2.40 cm^3^ (IQR 0.80 to 5.78). The other 8 subjects with no acute infarct on DWI were regarded as TIA. Twenty-four subjects had MCA stenosis, 11 had intracranial ICA stenosis, and 1 had tandem ICA-MCA stenosis. Twenty-three (63.9%) and 13 (36.1%) lesions were anatomically severe and moderate stenoses, respectively. All the 6 ICASs identified on 1.5 T MRA, and 17 of the 30 lesions identified on 3.0 T MRA, were anatomically severe lesions. Overall, mean SIR was 0.84±0.23, with a range of 0.37 to 1.28.

### Associations between SIR values and baseline characteristics

**Figure 2:**
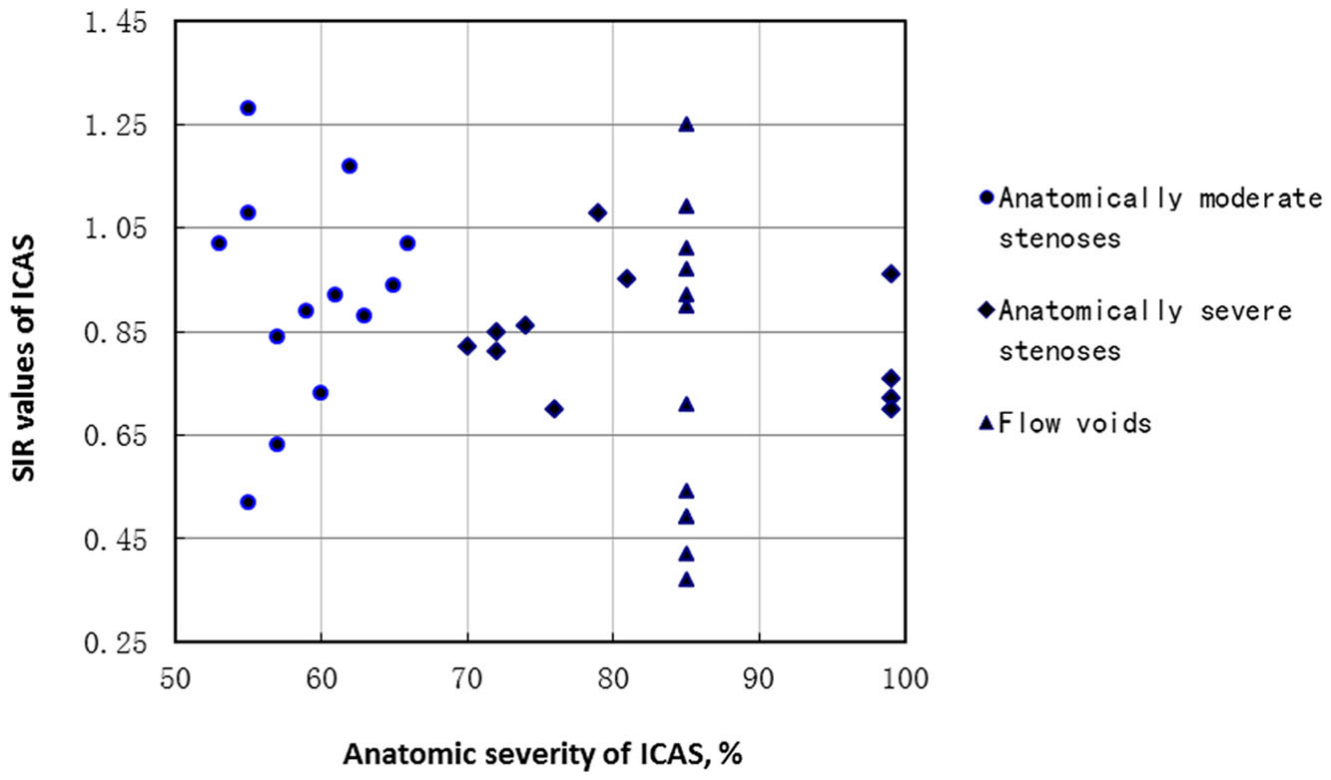
SIR values of anatomically moderate (dots) and severe (diamonds and triangles) stenoses. Flow voids on MRA were regarded as anatomically severe stenoses in this study and illustrated as 85% stenoses (triangles) in the scatterplot. SIR, signal intensity ratio; MRA, magnetic resonance angiography.

Mean SIRs were not significantly different between those with or without histories of dyslipidemia, hypertension, diabetes mellitus, ischemic heart disease and stroke/TIA. Overall, mean SIR of anatomically moderate stenoses tended to be higher than that of anatomically severe stenoses (0.92±0.21 versus 0.80±0.23), but not statistically significant (p = 0.126) (Table 2 and Figure 2), the trend of which was in accordance with the comparison of SIR values of anatomically moderate and severe lesions (0.92±0.21 versus 0.86±0.21, p = 0.439) among the 30 ICASs detected on 3.0 T MRA.

**Table 2:** Comparison of SIR values.

	Mean SIR value a		P value for Mann-
Characteristics	Yes	No	Whitney test
Male	0.83 (0.22)	0.93 (0.26)	0.207^b^
Severe stenosis	0.80 (0.23)	0.92 (0.21)	0.126^c^
Current smoker	0.83 (0.21)	0.86 (0.25)	0.788^c^
History of dyslipidemia	0.88 (0.25)	0.83 (0.22)	0.557^b^
History of hypertension	0.89 (0.22)	0.79 (0.23)	0.126^c^
History of diabetes mellitus	0.95 (0.23)	0.80 (0.21)	0.201^b^
History of ischemic heart disease	0.85 (0.16)	0.84 (0.24)	0.859^b^
History of stroke/TIA	0.92 (0.25)	0.82 (0.22)	0.287^b^
Ischemic stroke recurrence at 1-year	0.73 (0.30)	0.85 (0.23)	0.667^b^

SIR, signal intensity ratio; TIA, transient ischemic attack.

a Values are means (SD).

b Exact p values (2-tailed).

c Asymptotic p values based on the normal approximation (2-tailed).

**Figure 3:**
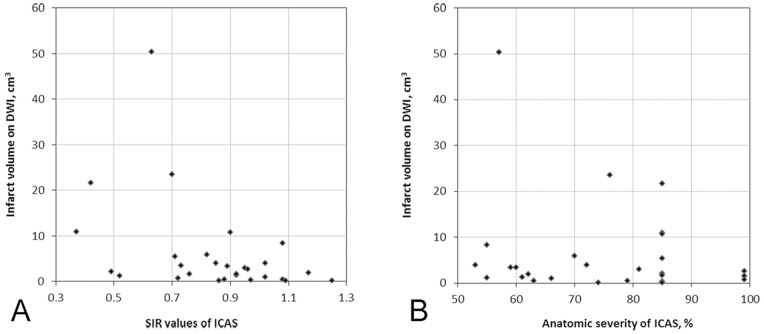
Relationships between SIR/anatomic severity of ICAS on MRA and acute infarct volume on DWI. A. Acute infarct volume on DWI was significantly, linearly and negatively correlated to SIR values of ICAS (p = 0.011); B. Anatomic severity of ICAS on MRA and infarct volume on DWI were not significantly linearly correlated (p = 0.360). SIR, signal intensity ratio; ICAS, intracranial arterial stenosis; MRA, magnetic resonance angiography; DWI, diffusion-weighted imaging.

For the 28 ischemic stroke patients, Spearman correlation coefficient of SIR and infarct volume on DWI was −0.471 (p = 0.011). Thus, SIR and infarct volume was significantly, linearly and negatively correlated (Figure 3.A). However, the anatomic severity of ICAS was not significantly linearly related to infarct volume on DWI (Spearman correlation coefficient −0.180, p = 0.360; Figure 3.B). Among the 28 ischemic stroke patients, the patient with the largest acute infarct volume of 50.33 cm^3^, is shown as an outlier on Figure 3 (A and B). The correlation between SIR values and infarct volumes (Spearman correlation coefficient −0.433, p = 0.024), as well as that between the anatomic severity of ICAS and infarct volumes (Spearman correlation coefficient −0.104, p = 0.606), was the same, when the outlier was excluded from analyses, as compared with analyses based on the 28 ischemic stroke patients.

### Associations between SIR values and 1-year outcome

**Figure 4:**
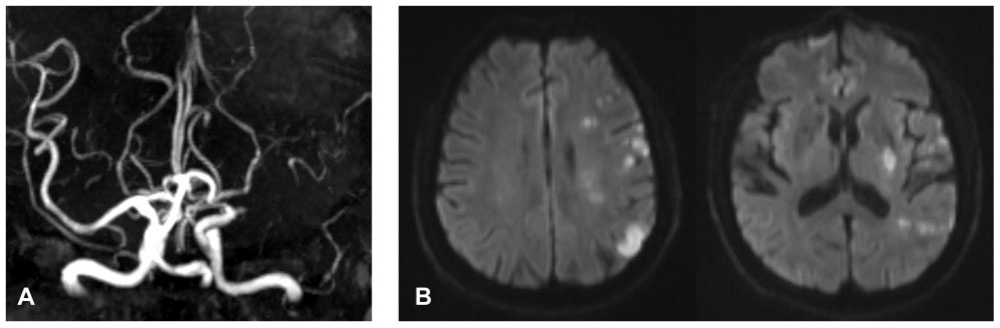
Endpoint MRA and DWI images of one of the patients with recurrent ischemic stroke, who had a SIR of 0.95 for left MCA stenosis at baseline. MRA (A) showed left MCA occlusion and DWI (B) showed multiple and small subcortical/cortical lesions within left MCA territory. MRA, magnetic resonance angiography; DWI, diffusion-weighted imaging; SIR, signal intensity ratio; MCA, middle cerebral artery.

At 1-year follow-up, 2 (5.6%) patients were reported to reach the primary endpoint, both were nonfatal recurrent ischemic stroke. No secondary endpoint events were reported. No baseline variable was found to be significantly different between patients with or without recurrent ischemic stroke, including the mean SIR value. The baseline SIR values of the two patients with recurrent ischemic stroke were 0.95 for a left MCA lesion and 0.52 for a right MCA lesion, respectively. The one with a baseline SIR of 0.95 underwent MRA and DWI at stroke recurrence, which showed left MCA occlusion and multiple subcortical and cortical lesions within left MCA territory (Figure 4). Without any potential evidence supporting a cardioembolic stroke, the mechanism of the recurrent event was most likely to be artery-to-artery embolism, though the origin of potential emboli was unknown. The other patient with recurrent stroke was followed up by phone, hence no detailed imaging information for the endpoint event.

## Discussion

In this preliminary study on MRA signal intensity and hemodynamic impairment of intracranial arterial stenosis, we found that mean SIRs were not significantly different between anatomically severe and moderate stenoses. We also demonstrated that SIR values were significantly correlated with infarct volumes at baseline in ischemic stroke patients. However, we did not establish a definite correlation between SIR and 1-year outcome among the 36 subjects recruited.

Anatomically severe symptomatic stenosis (70–99%) on catheter angiograms was found to be related to higher risk of subsequent stroke in the WASID study [14], and it has drawn a lot of attention in clinical practice as well as in clinical trials of intracranial atherosclerosis. For instance, the Stenting versus Aggressive Medical Therapy for Intracranial Arterial Stenosis (SAMMPRIS) trial focused only on patients with severe ICAS [15]. This emphasis does not suggest that patients with anatomically moderate stenosis, a large population in Asia, would have no risk for stroke. According to the results in our study, SIRs of subjects with severe stenosis tended to be lower than those with moderate stenosis (p = 0.126), suggesting that mostly anatomically severe stenoses may be more hemodynamically significant, compared with moderate stenoses. On the other hand, since the trend was not statistically significant, it also implied that at least some of the anatomically moderate stenoses may be hemodynamically significant, as shown in Figure 2 with very low SIRs. This may partly explain why some patients with anatomically moderate ICAS also develop subsequent ischemic stroke. Different field strengths may not interfere with this trend identified in the 36 cases recruited in the current study, due to the findings that there was no significant difference in mean SIR values of anatomically severe and moderate stenoses in the 30 lesions detected by 3.0 T MRA, either. However, generalization of conclusions from the current study may be limited by the small sample size. Larger studies such as ours may further clarify this potential disparity between anatomic and hemodynamic severity of ICAS and show that hemodynamic significance of ICAS should be evaluated, otherwise patients with anatomically moderate but hemodynamically severe stenosis could be underdiagnosed and/or undertreated, and vice versa.

Based on the mechanism of TOF MRA, lower SIR indicates impaired flow and perfusion distal to the stenosis. In other word, SIR measured on MRA MIPs could to some extent reflect functional severity of intracranial arterial stenosis. This hypothesis was supported by our findings that SIR rather than percentage stenosis of ICAS was significantly correlated to DWI infarct volume: subjects with lower SIR values had larger infarct volumes, which correlations were not altered after excluding the subject with the largest infarct volume on DWI, an outlier on Figure 3 (A and B). The role of SIR in reflecting distal flow in the case of ICAS could be further verified in future studies by investigating relationships between SIR and distal brain perfusion, using relevant imaging modalities, for instance, perfusion-weighted imaging and arterial spin labeling, etc.

We had hypothesized that baseline SIR could have predictive value for outcomes. However, we failed to establish such relationships between baseline SIR and 1y outcomes in the current study, probably due to the small sample size and the low rate of stroke recurrence (5.6%). Of the 2 patients with primary endpoints, only one had neuroimaging results at recurrence, the most likely cause being artery-to-artery embolism, which is an important mechanism of cerebral infarcts in patients with MCA stenosis [16]. So this recurrent stroke could be due to unstability of plaques in situ or in proximal arterial segments, another aspect of ICAS that should be paid attention to, and may have nothing to do with the functional severity of the stenosis itself, represented as SIR in this study. Recently, a larger study performed in 189 patients with symptomatic ICAS in the Stroke Outcomes and Neuroimaging of Intracranial Atherosclerosis (SONIA) trial [17] found that a SIR <0.9 was independently related to recurrent stroke in the territory of the symptomatic artery (hazard ratio 10.9; 95% CI 2.0–58.9; p = 0.001) [18]. Therefore, despite of the negative findings on SIR and 1y outcomes in the present study, the ability of SIR to predict prognosis of patients with symptomatic ICAS still warrants further investigation.

This study had some limitations, among which the small sample size as mentioned above was the most important one. The number of primary endpoint events was too small to test our hypothesis on SIR and outcomes of ischemic stroke or TIA patients with ICAS. Besides, though we have demonstrated the SIR index to be reproducible between observers when used to evaluate ICAS located at the trunk of major intracranial arteries [10], there exist technical limitations with 3D TOF MRA, regarding signal measurements at different locations in the intracranial vasculature, which possibly yield unreliability for measurements of SIR for ICAS located at branches of major intracranial arteries that were not covered in our relevant studies. Partly due to this reason, we only recruited patients with symptomatic single vessel disease within the anterior circulation in this preliminary study, while the highly selective study population were not representative enough for the generalization of the study findings. Future studies could be performed in larger sample sizes, multiple vessel diseases, or ICAS in anterior or posterior circulations, to further validate our findings.

## Conclusions

Change of signal intensities across an intracranial arterial stenosis on MRA may reflect functional and hemodynamic severity of the lesion. Future studies are warranted to further verify the relationships between this index and short- and/or long-term prognosis of patients with symptomatic ICAS.

## References

[1] Wong LKS (2006) Global burden of intracranial atherosclerosis. Int J Stroke 1: 158–159. 18706036 10.1111/j.1747-4949.2006.00045.x

[2] Huang YN, Gao S, Li SW, Huang Y, Li JF, et al (1997) Vascular lesions in Chinese patients with transient ischemic attacks. Neurology 48: 524–525. 9040750 10.1212/wnl.48.2.524

[3] Liu HM, Tu YK, Yip PK, Su CT (1996) Evaluation of intracranial and extracranial carotid steno-occlusive diseases in Taiwan Chinese patients with MR angiography: preliminary experience. Stroke 27: 650–653. 8614924 10.1161/01.str.27.4.650

[4] Wong KS, Huang YN, Gao S, Lam WW, Chan YL, et al (1998) Intracranial stenosis in Chinese patients with acute stroke. Neurology 50: 812–813. 9521286 10.1212/wnl.50.3.812

[5] Wong KS, Li H, Chan YL, Ahuja A, Lam WWM, et al (2000) Use of transcranial Doppler ultrasound to predict outcome in patients with intracranial large-artery occlusive disease. Stroke 31: 2641–2647. 11062288 10.1161/01.str.31.11.2641

[6] Qureshi AI, Feldmann E, Gomez CR, Johnston SC, Kasner SE, et al (2009) Intracranial atherosclerotic disease: an update. Ann Neurol 66: 730–738. 20035502 10.1002/ana.21768

[7] Bradley WG, Waluch V, Lai KS, Fernandez EJ, Spalter C (1984) The appearance of rapidly flowing blood on magnetic-resonance images. Am J Roentgenol 143: 1167–1174. 6333786 10.2214/ajr.143.6.1167

[8] Mustert BR, Williams DM, Prince MR (1998) In vitro model of arterial stenosis: Correlation of MR signal dephasing and trans-stenotic pressure gradients. Magn Reson Imaging 16: 301–310. 9621971 10.1016/s0730-725x(97)00304-4

[9] Leng X, Wong LK, Soo Y, Leung T, Zou X, et al (2013) Signal intensity ratio as a novel measure of hemodynamic significance for intracranial atherosclerosis. Letter to the Editor. Int J Stroke 8: E46. 24024922 10.1111/ijs.12080 PMC4156586

[10] Leng X, Ip HL, Soo Y, Leung T, Liu L, et al.. (2013) Inter-observer reproducibility of signal intensity ratio on MR angiography for hemodynamic impact of intracranial atherosclerosis. J Stroke Cerebrovasc Dis: 2013 Sep 25. Epub ahead of print. 10.1016/j.jstrokecerebrovasdis.2013.07.036 PMC3873834 24075586

[11] Qian Y, Pu Y, Liu L, Wang DZ, Zhao X, et al (2013) Low HDL-C level is associated with the development of intracranial artery stenosis: analysis from the Chinese IntraCranial AtheroSclerosis (CICAS) study. PLoS One 8: e64395. 23691210 10.1371/journal.pone.0064395 PMC3656851

[12] Easton JD, Saver JL, Albers GW, Alberts MJ, Chaturvedi S, et al (2009) Definition and evaluation of transient ischemic attack: a scientific statement for healthcare professionals from the American Heart Association/American Stroke Association Stroke Council; Council on Cardiovascular Surgery and Anesthesia; Council on Cardiovascular Radiology and Intervention; Council on Cardiovascular Nursing; and the Interdisciplinary Council on Peripheral Vascular Disease. Stroke 40: 2276–2293. 19423857 10.1161/STROKEAHA.108.192218

[13] Samuels OB, Joseph GJ, Lynn MJ, Smith HA, Chimowitz MZ (2000) A standardized method for measuring intracranial arterial stenosis. Am J Neuroradiol 21: 643–646. 10782772 PMC7976653

[14] Kasner SE, Chimowitz MI, Lynn MJ, Howlett-Smith H, Stern BJ, et al (2006) Predictors of ischemic stroke in the territory of a symptomatic intracranial arterial stenosis. Circulation 113: 555–563. 16432056 10.1161/CIRCULATIONAHA.105.578229

[15] Chimowitz MI, Lynn MJ, Derdeyn CP, Turan TN, Fiorella D, et al (2011) Stenting versus Aggressive Medical Therapy for Intracranial Arterial Stenosis. N Engl J Med 365: 993–1003. 21899409 10.1056/NEJMoa1105335 PMC3552515

[16] Wong KS, Gao S, Chan YL, Hansberg T, Lam WWM, et al (2002) Mechanisms of acute cerebral infarctions in patients with middle cerebral artery stenosis: A diffusion-weighted imaging and microemboli monitoring study. Ann Neurol 52: 74–81. 12112050 10.1002/ana.10250

[17] The SONIA Trial Investigators (2004) Stroke Outcome and Neuroimaging of Intracranial Atherosclerosis (SONIA): design of a prospective, multicenter trial of diagnostic tests. Neuroepidemiology 23: 23–32. 14765534 10.1159/000073971

[18] Liebeskind DS, Kosinski AS, Lynn MJ, Scalzo F, Fong AK, et al (2013) Noninvasive fractional flow on MRA predicts stroke risk of intracranial stenosis in SONIA/WASID. Stroke 44: ATP166. 10.1111/jon.12101 PMC4156566 24593693

